# Attenuation correction for hybrid MR/PET scanners: a comparison study

**DOI:** 10.1186/2197-7364-2-S1-A38

**Published:** 2015-05-18

**Authors:** Elena Rota Kops, Andre Santos Ribeiro, Liliana Caldeira, Hubertus Hautzel, Mathias Lukas, Gerald Antoch, Christoph Lerche, Jon Shah

**Affiliations:** Forschungszentrum Jülich GmbH, Jülich, Germany; Imperial College London, London, UK; Heinrich-Heine-University Düsseldorf, Düsseldorf, Germany; Technische Universitaet Muenchen, Munich, Germany

Attenuation correction of PET data acquired in hybrid MR/PET scanners is still a challenge. Different methods have been adopted by several groups to obtain reliable attenuation maps (mu-maps). In this study we compare three methods: MGH, UCL, Neural-Network. The MGH method is based on an MR/CT template obtained with the SPM8 software. The UCL method uses a database of MR/CT pairs. Both generate mu-maps from MP-RAGE images. The feed-forward neural-network from Juelich (NN-Juelich) requires two UTE images; it generates segmented mu-maps. Data from eight subjects (S1-S8) measured in the Siemens 3T MR-BrainPET scanner were used. Corresponding CT images were acquired. The resulting mu-maps were compared against the CT-based mu-maps for each subject and method. Overlapped voxels and Dice similarity coefficients, D, for bone, soft-tissue and air regions, and relative differences images were calculated. The true positive (TP) recognized voxels for the whole head were 79.9% (NN-Juelich, S7) to 92.1% (UCL method, S1). D values of the bone were D=0.65 (NN-Juelich, S1) to D=0.87 (UCL method, S1). For S8 the MHG method failed (TP=76.4%; D=0.46 for bone). D values shared a common tendency in all subjects and methods to recognize soft-tissue as bone. The relative difference images showed a variation of -10.9% - +10.1%; for S8 and MHG method the values were -24.5% and +14.2%. A preliminary comparison of three methods for generation of mu-maps for MR/PET scanners is presented. The continuous methods (MGH, UCL) seem to generate reliable mu-maps, whilst the binary method seems to need further improvement. Future work will include more subjects, the reconstruction of corresponding PET data and their comparison.

